# A randomized controlled trial of shared decision-making treatment planning process to enhance shared decision-making in patients with MBC

**DOI:** 10.1007/s10549-024-07304-y

**Published:** 2024-06-10

**Authors:** Gabrielle B. Rocque, Noon Eltoum, Nicole E. Caston, Courtney P. Williams, Marian M. Oliver, Lauren Moradi, Stacey Ingram, Andres Azuero, Maria Pisu, Smita Bhatia

**Affiliations:** 1 O’Neal Comprehensive Cancer Center, University of Alabama at Birmingham, Birmingham, AL 35294 USA; 2 Division of Hematology and Oncology, Department of Medicine, University of Alabama at Birmingham, Birmingham, AL 35294 USA; 3 Division of Gerontology/Geriatrics/Palliative Care, Department of Medicine, University of Alabama at Birmingham, South, AL 35294 USA; 4 Division of Preventive Medicine, University of Alabama at Birmingham, Birmingham, AL USA; 5 School of Nursing, University of Alabama at Birmingham, Birmingham, AL USA; 6 Institute for Cancer Outcomes and Survivorship, University of Alabama at Birmingham, Birmingham, AL USA

**Keywords:** Shared decision-making, Treatment planning, Patient-reported data, Metastatic breast cancer

## Abstract

**Purpose:**

Opportunities exist for patients with metastatic breast cancer (MBC) to engage in shared decision-making (SDM). Presenting patient-reported data, including patient treatment preferences, to oncologists before or during a treatment plan decision may improve patient engagement in treatment decisions.

**Methods:**

This randomized controlled trial evaluated the standard-of-care treatment planning process vs. a novel treatment planning process focused on SDM, which included oncologist review of patient-reported treatment preferences, prior to or during treatment decisions among women with MBC. The primary outcome was patient perception of shared decision-making. Secondary outcomes included patient activation, treatment satisfaction, physician perception of treatment decision-making, and use of treatment plans.

**Results:**

Among the 109 evaluable patients from December 2018 to June 2022, 28% were Black and 12% lived in a highly disadvantaged neighborhood. Although not reaching statistical significance, patients in the intervention arm perceived SDM more often than patients in the control arm (63% vs. 59%; Cramer’s *V* = 0.05; OR 1.19; 95% CI 0.55–2.57). Among patients in the intervention arm, 31% were at the highest level of patient activation compared to 19% of those in the control arm (*V *= 0.18). In 82% of decisions, the oncologist agreed that the patient-reported data helped them engage in SDM. In 45% of decision, they reported changing management due to patient-reported data.

**Conclusions:**

Oncologist engagement in the treatment planning process, with oncologist review of patient-reported data, is a promising approach to improve patient participation in treatment decisions which should be tested in larger studies.

**Trial registration:**

NCT03806738.

**Supplementary Information:**

The online version contains supplementary material available at 10.1007/s10549-024-07304-y.

## Introduction

A unique opportunity exists for patients with metastatic breast cancer (MBC) to engage in shared decision-making (SDM) due to the over 50 MBC treatment regimens (single drug or combination) included in the National Comprehensive Cancer Network guidelines [1], the sequential nature of systemic treatment over the course of a patient’s illness, and the varying toxicity profiles for different treatment regimens [2, 3]. Furthermore, given the incurable nature of MBC, individuals may have different treatment priorities and preferences (i.e., maximizing survival vs. quality of life).

Though most patients with cancer desire an active or shared role in treatment decision-making [4–6], 40% of patients experience discordance between their preferred and actual decision-making roles, with most reporting less than desired involvement [6]. Our prior qualitative work demonstrated that oncologists’ treatment selection for MBC is prominently driven by treatment efficacy and physical side effects [7]. However, treatment factors considered by patients were more varied, and included treatment cost, logistics, side effects, salience of cutting edge treatments, and impact on personal responsibilities, important life events, and daily living [7, 8]. Discussions of preferences are uncommon since few avenues exist to capture and discuss treatment preferences due to numerous barriers including workflow challenges, limited clinician time, and misaligned incentives [9]. In one study by Williams et al., 67% of oncologists reported that patients’ personal quality of life priorities and preferences had a major impact on treatment recommendations, yet only 37% of patients reported having discussions about priorities and preferences prior to treatment selection [10].

Black patients may face additional challenges engaging in decisions. Black women report feeling less prepared for engaging in decisions or experience discomfort taking an active role when talking with physicians [11]. This is consistent with our prior study in which more Black women than White women with MBC preferred physician-led decision-making (32% vs. 28%) [12]. This may be related, in part, to differential patient activation, which includes the knowledge, skill, confidence, and ability to engage with the medical team to be able to take an active role in healthcare. Black patients have overall lower levels of patient activation, but higher activation moderates the relationship between implicit bias and patient-perceived quality of care [13]. Thus, consideration of this subpopulation is critical when evaluating SDM interventions.

One approach to enhance SDM is to incorporate patient-reported data into the process for generating treatment plans, which are lay language documents which the oncology team provides to patients, caregivers, and other healthcare providers that summarize the patient’s diagnosis, planned treatments, and potential side effects [14]. In early-stage breast cancer, treatment plans improved patient knowledge about their cancer and treatments, facilitated patient–clinician communication, enhanced coordination of care, and helped patients feel prepared for cancer treatment [15, 16]. However, these early studies did not leverage the use of patient-reported data. Prior literature highlights improvements in communication between oncologists, patients, and caregivers, as well as ability to identify better patient needs during treatment [17]. Thus, inclusion of patient-reported outcomes within the treatment planning process represents an opportunity to activate patients and increase shared decision-making.

Within our institution, standard-of-care treatment plans include patient-reported data (e.g., distress assessment, illness understanding, interest in clinical trials, presence of advance directives, preferred locus of control for decisions) [18]. Treatment plans are delivered by nurses after treatment is started to reinforce education, and the patient-reported treatment planning data are rarely reviewed by oncologists prior to treatment decisions. We hypothesized that presenting patient-reported data, including treatment preferences, to oncologists before or during a treatment plan decision would improve patient activation and shared decision-making. To this end, we conducted a randomized controlled trial to test the impact on decision-making outcomes of a patient-reported data-driven, oncologist-engaged, SDM treatment planning process compared to the standard treatment planning.

## Methods

### Trial design

This parallel group randomized controlled trial evaluated the impact of a SDM treatment planning process on decision-making outcomes in women with MBC. This trial follows the Consolidated Standards of Reporting Trials (CONSORT) [19] and was approved by the Advarra Central Internal Review Board (IRB-300002283; NCT03806738).

### Participants

Research coordinators screened clinic lists for women receiving MBC treatment at the O’Neal Comprehensive Cancer Center at the University of Alabama at Birmingham (UAB). Patients aged 18 years or older making any treatment decision, including initial or later lines of therapy, were eligible for inclusion. Exclusion criteria included patients unable to read and/or speak English, patients not receiving therapy at UAB, patients with life expectancy <3 months, and patients unable to provide informed consent. Patients provided written or verbal informed consent to participate.

### Treatment decisions

Treatment decisions considered in this study related to medical therapy (e.g., chemotherapy, targeted therapy, hormonal therapy), including changing therapy or discontinuing treatment.

### Control

As part of standard-of-care, UAB housed a digital platform (Carevive^®^) which merges (1) questionnaire-based patient-reported data (Appendix A) and (2) clinical data from the electronic medical record to generate a patient-centric treatment planning document that met the requirements of the Oncology Care Model treatment plan [20]. Notably, the patient-reported data did not include patient treatment preferences. To capture updates in patient-reported data, patients were surveyed every 6 months until the treatment decision was made. The oncologists did not routinely review the patient-reported data. This treatment planning document was delivered by the nurse after the treatment decision and used as an education tool approximately 1 month after the decision (after the post-decision survey).

### Intervention

**Fig. 1:**
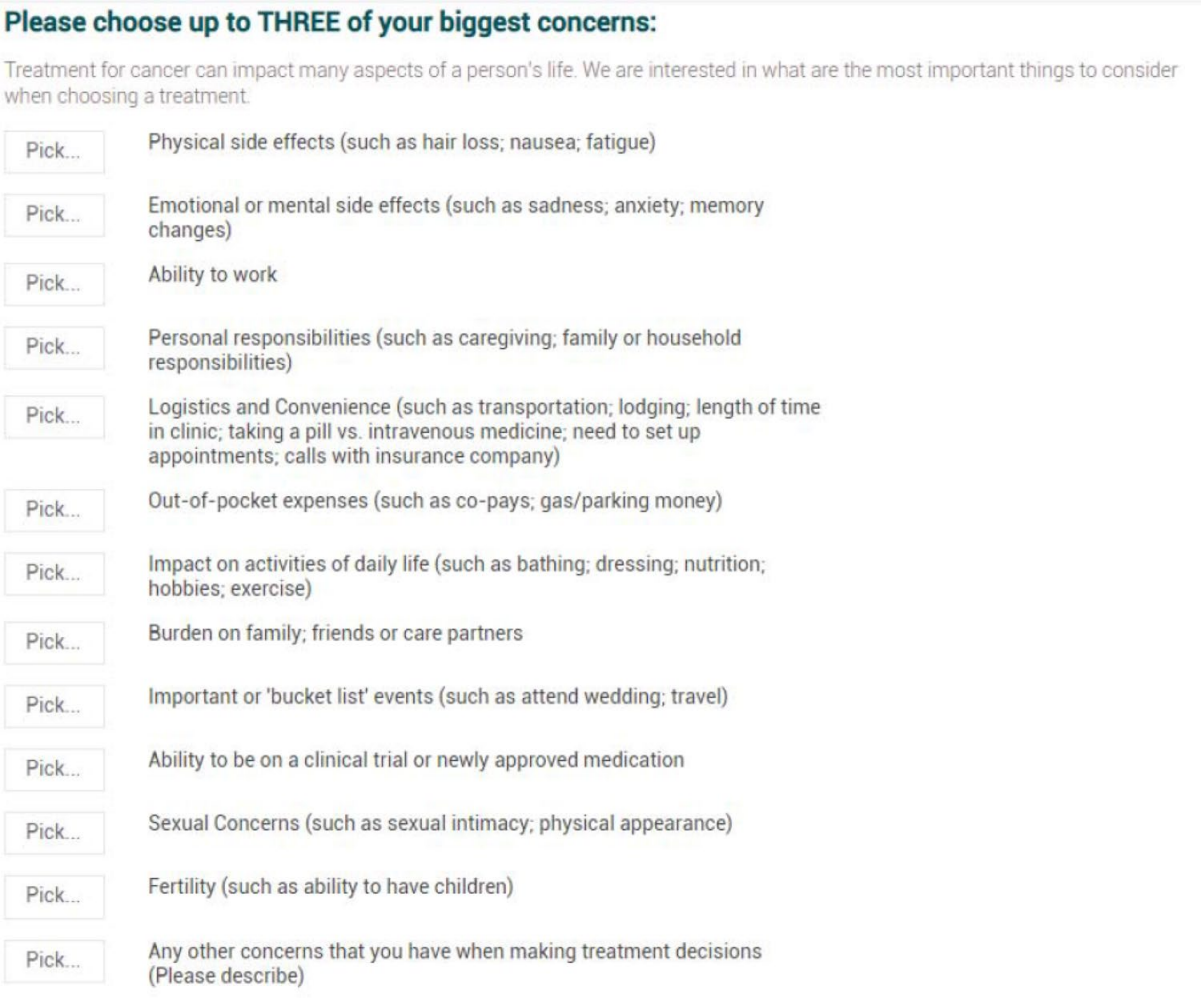
Preference question used within the treatment plan

Patients randomized to the intervention arm received three key distinctions from the standard treatment planning process. First, the treatment planning survey included a treatment preference elicitation questionnaire (Fig. 1) [21]. Second, a research coordinator reviewed the patient-reported data with oncologists before or during the next treatment decision. Third, the treatment plan was delivered to patients randomized to the intervention during the treatment decision or shortly afterward (prior to post-decision survey).

### Primary outcome

The primary outcome was patient *perception of* SDM captured 1 month after the treatment decision, measured using the Control Preferences Scale [5, 22]. Responses were grouped into three decision-making categories, including patient-driven (“I made the final selection about which treatment I would receive”; “I made the final selection of my treatment after seriously considering my doctor’s opinion.”), shared (“My doctor and I shared responsibility for deciding which treatment was best for me.”), or provider-driven (“My doctor made the final decision about which treatment will be used but seriously considered my opinion”; “My doctor made all the decisions regarding my treatment.”).

### Secondary outcomes

*Patient-reported:* Secondary outcomes assessed after a patient made a treatment decision included patient activation using the Patient Activation Measure (PAM) [23]. PAM scores range from 0 to 100 and fall under four levels of activation with higher levels indicating higher activation [24]. Other secondary endpoints included patient treatment satisfaction using the Treatment Satisfaction Questionnaire for Medication (TSQM), which is a 14-item questionnaire that consists of four subscales that measure effectiveness, side effects, convenience, and global satisfaction [25]. Each subscale score ranges from 0 to 100, where higher scores represent more satisfaction with medication/treatment. The Consumer Assessment of Healthcare Providers and Systems (CAHPS) SDM questionnaire was used to assess SDM [26] using the top box scoring methodology [27]. For this questionnaire, answers of “yes, definitely” were compared against “yes, somewhat” and “no.” Using the Control Preferences Scale [5], patient *preference for* decision-making was assessed at baseline before a treatment decision was made. Concordance between baseline *preference for* (Appendix A) and post-decision *perception of* decision-making role was evaluated using the Control Preferences Scale [5] (Appendix B).

*Oncologist-reported:* Secondary outcomes included oncologist perception of decision-making (shared vs. patient- or oncologist-centric) and concordance between the oncologist and patient perception of decision-making (Control Preferences Scale [5]). For patients receiving the intervention, oncologist use of the treatment plan, change in patient treatment management based on patient-reported data, and perception of treatment plan utility to engage in SDM were captured immediately following the treatment decision (Appendix C).

### Participant characteristics

Patient age, race and ethnicity, address, insurance status, MBC type (de novo or recurrent), and receptor status were abstracted from the electronic medical record. Area Deprivation Index (ADI), a validated measure of socioeconomic disadvantage, was used to determine patient-level neighborhood disadvantage [28]. ADI is scored from 0 to 100, with higher scores indicating greater disadvantage. Rural–Urban Commuting Area Codes (RUCA) were used to determine the level of rurality or urbanicity of a patient’s residence [29]. Distance and time traveled for care were calculated from patient home to main UAB campus [30]. Desire for clinical trial enrollment was self-reported and collected from patient survey responses.

### Randomization

Consenting participants were randomly assigned in a 1:1 allocation ratio to receive the intervention or standard-of-care using SAS© software, version 9.4 (SAS Institute, Cary, NC)-generated, permuted block randomization stratified by oncologist [31]. Sealed envelopes were used to blind assignment before randomization, so study personnel were blinded pre-randomization. Participants and study personnel were not blinded post-randomization.

### Power and sample size

Assuming a 50% percentage of SDM perception in the control group, based on 80% power and 0.05 significance level, a sample size of 140 patients (70 patients per arm) making a decision is required to detect an estimated 20% between-group difference in perception of SDM.

### Statistical methods

Descriptive statistics were calculated using frequencies and percentages for categorical data. Between-group balance was examined using measures of effect size of descriptive characteristics between arms: Cramer’s V (*V* = 0.1 indicates small effect size, *V* = 0.3 indicates medium effect size, and *V* = 0.5 indicates large effect size [32]). For age, the median and interquartile range (IQR) were calculated, and Cohen’s *d* was used to assess effect size (*d* = 0.2 indicates small effect size, *d* = 0.5 indicates medium effect size, and *d* = 0.8 indicates large effect size [32]). For other continuous variables, median and IQR were calculated, and Kendall τb rank correlation coefficient was used to assess the magnitude of between-group differences (τb = 0.1 indicates small effect size, τb = 0.3 indicates medium effect size, and τb = 0.5 indicates large effect size [33]). Using binomial logistic regression models, odds ratios (OR) and 95% confidence intervals were estimated to evaluate the intervention effect on patient’s perception of decision-making. Alpha level was set at 0.05. Per-protocol and intention-to-treat analyses (Supplemental Appendix Table 1) were performed using SAS^®^ software, version 9.4 (SAS Institute, Cary, NC). An exploratory subset analysis of decision-making perception and patient activation was conducted for Black patients, who were hypothesized to have increased ability to benefit from preference evaluation due to previous reports of lower communication quality [34].

## Results

### Patient characteristics

**Fig. 2:**
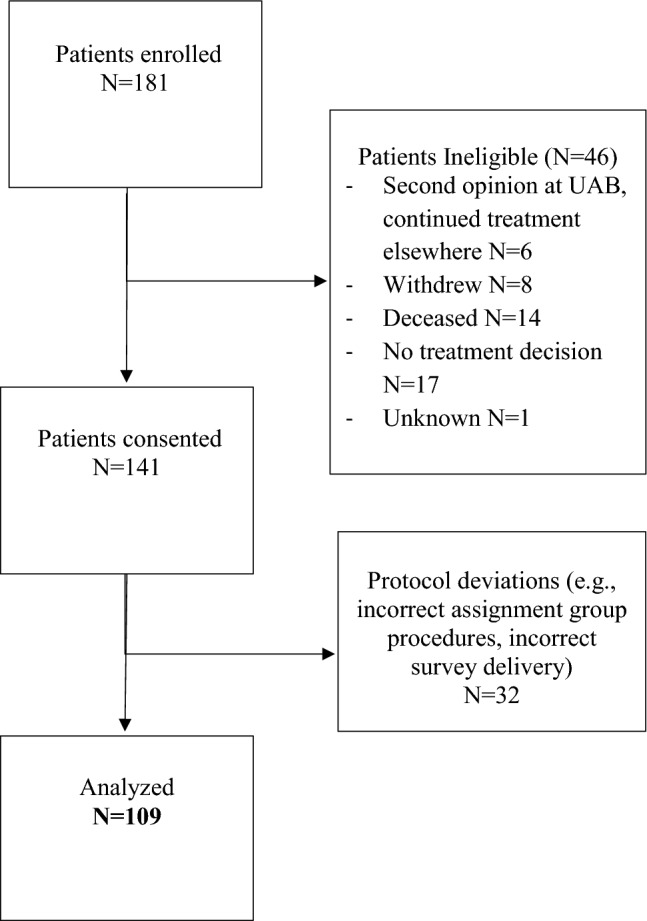
CONSORT diagram

Overall, 187 patients were enrolled from December 2018 to June 2022; 141 patients had a treatment decision at the time of study closure; 32 patients were deemed not evaluable; and 109 patients were included in the final analysis (Fig. 2 and Table 1). Patients included and excluded from the analysis were similar, except patients excluded were slightly older (Supplemental material Table 2). Of included patients (*N* = 109), median age at consent was 59 (IQR 49–65), 28% were Black, 12% lived in a highly disadvantaged neighborhood (ADI > 15%), and 24% lived in a rural setting. There was a total of 51 patients randomized to the intervention and 58 to the control. At baseline, most patients preferred a shared (57%) or a patient-centric (34%) approach to decision-making. Baseline characteristics were balanced between randomization arms, with a moderate imbalance in cancer subtype (Table 1).

**Table 1:** Demographic and clinical characteristics of patients by study arm (*N* = 109)

	Total (*N* = 109)	Intervention (*n* = 51)	Control (*n* = 58)	Cramer’s V
	*n* (%)			
*Age at consent Median (IQR)*	59 (49–65)	60 (48–66)	59 (51–65)	Cohen’s *d* = 0.07
*Race and ethnicity*				0.11
Asian	2 (1.8)	1 (2.0)	1 (1.7)	
Black	30 (27.5)	13 (25.5)	17 (29.3)	
Hispanic/Latino	1 (0.9)	1 (2.0)	0	
White	74 (67.9)	35 (68.6)	39 (67.2)	
Declined	2 (1.8)	1 (2.0)	1 (1.7)	
*Area Deprivation Index (ADI)*				0.06
Least disadvantaged	57 (52.3)	27 (52.9)	30 (51.7)	
Most disadvantaged	13 (11.9)	7 (13.7)	6 (10.3)	
Unknown	39 (35.8)	17 (33.3)	22 (37.9)	
*Rural–Urban Commuting Area (RUCA)*				0.05
Rural	26 (23.9)	13 (25.5)	13 (22.4)	
Urban	73 (67.0)	34 (66.7)	39 (67.2)	
Unknown	10 (9.2)	4 (7.8)	6 (10.3)	
*Median time traveled (in minutes) (IQR)*	60 (30–99)	56 (26–82)	63 (31–102)	Kendall τb = −0.05
*Median distance traveled (in miles) Median (IQR)*	59 (24–99)	55 (20–89)	61 (24–106)	Kendall τb = 0.07
*Insurance status*				0.05
Private	39 (35.8)	18 (35.3)	21 (36.2)	
Medicaid	10 (9.2)	4 (7.8)	6 (10.3)	
Medicare	60 (55.1)	29 (56.9)	31 (53.5)	
*Cancer subtype*				0.22
HR+HER2+	22 (20.2)	7 (13.7)	15 (25.9)	
HR+HER2−	73 (67.0)	38 (74.5)	35 (60.3)	
HR−HER2+	4 (3.7)	3 (5.9)	1 (1.7)	
TNBC	10 (9.2)	3 (5.9)	7 (12.1)	
*Type of MBC*				0.12
De novo	8 (7.3)	2 (3.9)	6 (10.3)	
Recurrent	101 (92.7)	49 (96.1)	52 (89.7)	
*Patients desire for clinical trials*				0.05
Yes	92 (84.4)	44 (86.3)	48 (82.8)	
No	17 (15.6)	7 (13.7)	10 (17.2)	
*Baseline Control Preference Scale (Carevive)*				0.13
Oncologist-centric	9 (8.3)	4 (7.8)	5 (8.6)	
Shared	62 (56.9)	27 (52.9)	35 (60.3)	
Patient-centric	37 (33.9)	19 (37.3)	18 (31.0)	
N/A	1 (0.9)	1 (2.0)	0	

*HR* hormone receptor, *TNBC* triple-negative breast cancer, *IQR* interquartile range, *MBC* metastatic breast cancer, *N/A* not applicable, *HER2+* human epidermal growth factor receptor 2

### Primary outcome: shared decision-making

**Fig. 3:**
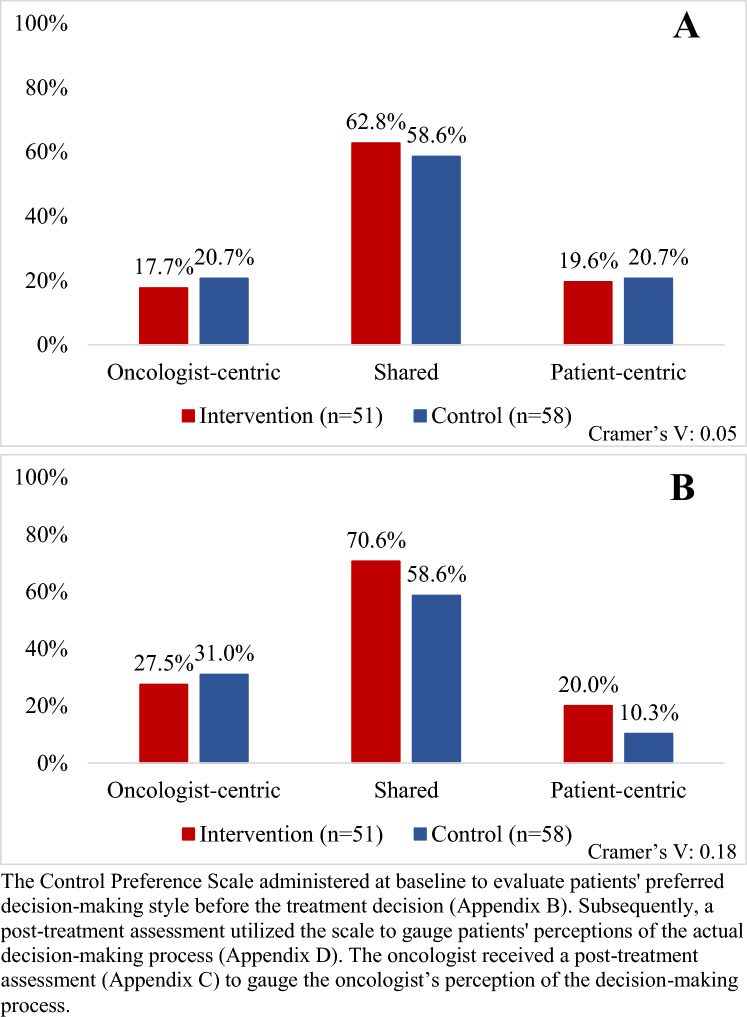
**A** Primary outcome: Patient perception of treatment decision-making using the Control Preferences Scale (*N* = 109). **B** Secondary outcome: Oncologist perception of treatment decision-making using the Control Preferences Scale (*N* = 109). Post-treatment assessment of patient and caregiver perceptions of how the decision was made were grouped into patient-centric, shared, and oncologist-centric

Patients in the intervention arm had 4% higher perception of SDM than patients in the control arm (63% vs. 59%; *V* = 0.05; Fig. 3A). The proportions of patients perceiving patient-centric (20% intervention vs. 21% control; *V* = 0.05) and oncologist-centric decision-making (18% intervention vs. 21% control; *V* = 0.05) were similar for patients in both arms. Patients in the intervention arm had 19% higher odds of perceiving SDM (OR 1.19; 95% CI 0.55–2.57), though not statistically significant. Among Black patients receiving the intervention, 77% perceived the treatment decision as shared, compared to 65% of Black patients in the control arm (*V* = 0.10).

### Secondary outcome: patient outcomes

**Fig. 4:**
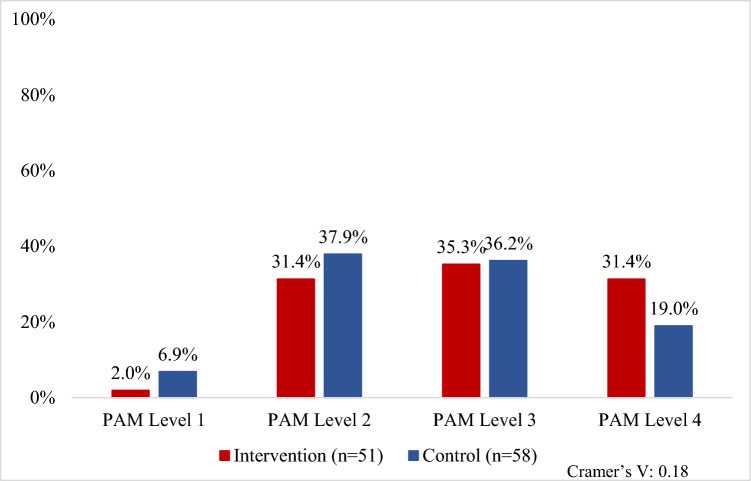
Patient Activation Measure scores by study arm (*N* = 109). Patient Activation Measure scores range from 0 to 100 and fall under four levels of activation. Level 1 implies low activation, or not believing activation is important. Level 2 indicates lack of knowledge and skills to take action. Level 3 implies beginning to take action, and level 4 is the highest level of activation and indicates a patient is proactive in managing their condition [24]

**Fig. 5:**
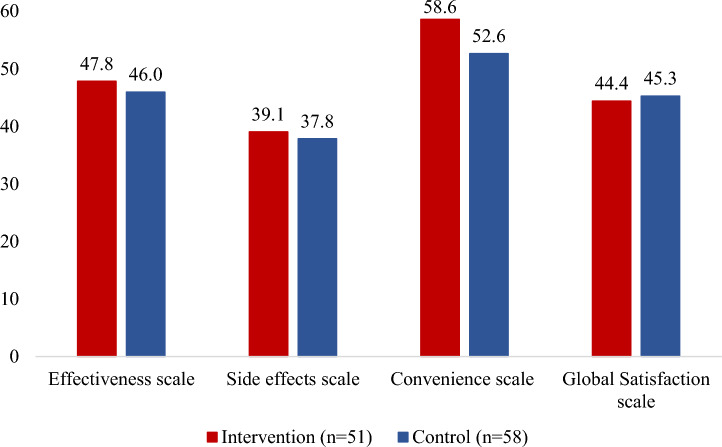
Average patient treatment satisfaction score for the subscales of the Treatment Satisfaction Questionnaire for Medication (*N* = 109). Treatment Satisfaction Questionnaire for Medication consists of the four subscales above. Each subscale score ranges from 0 to 100 where higher scores represent more satisfaction with medication/treatment

In the intervention arm, 31% were at the highest level of patient activation compared to 19% in the control arm (*V* = 0.18). Conversely, only 2% of patients in the intervention arm were at the lowest level of patient activation, compared to 7% in the control arm (Fig. 4). Mean treatment satisfaction scores were 6% higher in the intervention arm than the control arm for the convenience subscale (59 [SD = 16] vs. 53 [SD = 13]; *d* = 0.41). Although not significantly different, mean treatment effectiveness was slightly higher in the intervention arm than the control arm (48 [SD = 15] vs. 46 [SD = 12]; *d* = 0.10). Mean side effects subscales (39 [SD = 18] vs. 38 [SD = 19]; *d* = 0.07) and mean global satisfaction (*M* = 44 [SD = 19] vs. *M* = 45 [SD = 16]; *d* = 0.05; Fig. 5) were similar for both arms. Additionally, the proportions of CAHPS top box responses were similar between patients in the intervention and control arms (88% vs. 86%, *V* = 0.03). Concordance between patient *preference for* and *perception of* the decision-making was similar at 53% and 54% for patients in the intervention and control arms, respectively (*V* = 0.01).

### Secondary outcome: oncologist outcomes

Among oncologists, a higher proportion reported SDM perception for patients receiving the intervention compared to control (71% vs. 59%; *V* = 0.18). When not perceived to be shared, the decision was more often perceived to be oncologist-centric (28% intervention vs. 31% control) than patient-centric (2% intervention vs. 10% control; Fig. 3B). Additionally, the concordance between patient and oncologist perceptions of the decision was similar between arms (59% intervention vs. 60% control; *V* = 0.02). The oncologists reported using the patient-reported data and associated treatment plan during 88% of intervention decisions. The oncologist reported a change in management due to these data in 45% of decisions. The oncologist agreed that the patient-reported data helped them engage in SDM in 82% of decisions.

## Discussion

Although this randomized controlled trial in women with MBC did not meet the primary outcome with a statistically significant difference, the proportion of patients reporting SDM was 4% higher in the intervention arm than the control arm. In addition, non-significant, but numerically higher patient activation was observed for patients receiving the intervention compared to control patients (31% vs. 19% in highest activation level). Among Black patients receiving the intervention, 77% perceived the treatment decision as shared, compared to 65% of Black patients in the control arm. Finally, the favorable perception of the intervention from the oncologists and use in decision-making suggest that capture of patient preferences with subsequent review of patient-reported data as part of the decision-making process may enhance treatment decision-making and warrants further exploration.

Our study in patients with MBC complements existing studies on tools for SDM in early-stage breast cancer. In a study by Hawley and colleagues, the iCanDecide decision support tool demonstrated higher decision preparation and decision quality [35]. In another study of breast surgery decision aids, improvements in knowledge about treatment options, decisional conflict, and decision satisfaction were observed [36]. At the same time, these studies focused on specific decisions (e.g., mastectomy vs. lumpectomy) and providing knowledge to patients about those options. This differs from our study that provides a unique perspective on how oncologist review of patient-reported data can facilitate the incorporation of broader patient preferences and values into the treatment planning process.

In the majority of decisions, the oncologist used and valued the patient-reported data. This study adds to prior work by demonstrating how patient-reported data can be leveraged in clinical settings. For example, the geriatric assessment improves communication, reduces chemotherapy toxicities, and improves health-related quality of life compared to standard-of-care in older adults with cancer [37–41]. In other studies, patient-reported symptom monitoring with associated nurse management improved symptom assessment [42–44], patient–clinician communication and satisfaction [45, 46], and survival [47]. In aggregate, all of these studies highlight the opportunity for use of patient-reported data to better provide patient-centric care.

The greatest difference between the intervention and control group was observed in patient activation. Prior work demonstrates that higher activated patients are more likely to feel their treatment plans reflect their values, and they are more likely to tolerate side effects. In contrast, less activated patients have greater difficulty understanding their diagnosis and adhering to treatment [48]. We hypothesize that the discussion about their responses during the decision-making encounter contributed to the observed higher levels of patient activation among the intervention patients.

Inclusion of patient-reported preferences to guide decisions may be particularly important for patients whose voices may be historically unheard. One systematic review found that the majority of studies assessing patient–oncologist communication indicate that Black patients have lower quality patient–oncologist communication than White patients [34]. In a study of patients with lung cancer, Black patients viewed patient–oncologist communication to be less supportive than White patients [49]. In our study, more Black patients in the intervention arm perceived SDM (77%) than those in the control arm (65%). This may signal that a systematic approach to patient-reported data capture and review by oncologists could overcome known barriers in patient communication for vulnerable populations, which should be further explored.

**Fig. 6:**
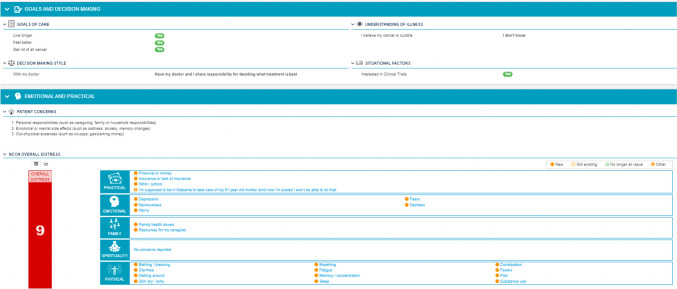
Example dashboard displaying patient preferences and other patient-reported data

The treatment plan process built upon for this intervention is designed to meet Oncology Care Model requirements, a requirement included in the current payment reform demonstration project, the Enhanced Oncology Model [50]. The novelty of this intervention is oncologist engagement through their review of electronic medical record-embedded patient dashboard and incorporation of patient-reported data into decision-making visits. The current version of the dashboard, which includes patient preferences, is shown in Fig. 6. Importantly, it does not require the oncologists to complete additional work to create or deliver treatment plans. The delegation of different healthcare team member roles (e.g., oncologist reviewing patient-reported data, nurse educating patient) remains important for the financial sustainability of the treatment planning process [51]. Also, focusing the oncologist role on review of patient-reported data likely contributed to the acceptability found in this study where the treatment planning process was noted to be helpful. In the setting of a busy oncology clinic, this structured approach of reviewing a simple patient-reported outcome dashboard may be more easily incorporated into existing workflows and overcome barriers related to limited oncologist training in preference elicitation. Future practice transformation efforts should carefully consider how to best engage oncologists in meaningful and efficient ways.

This study should be considered in light of several limitations. The study was conducted at a single academic medical center. There is potential for study contamination due to trained providers treating patients in both study arms. Such contamination would minimize observed impact. Furthermore, there was turnover and staffing shortages due to the COVID-19 pandemic. Sensitivity analysis accounting for time yielded similar results (not shown). When extended to the broader oncology community, use of the patient-reported data may differ between individuals. Additionally, this study focused specifically on patients with MBC, where there are multiple available treatment options, and thus it may not be applicable to patients with other cancer types, in the curative setting, or where treatment choices are limited. The patient-reported data also included multiple instruments in addition to the decision-related questions, and it is unknown which patient-reported data were most influential and longitudinal evaluation was not captured. Finally, although the target accrual of 140 decisions was met, the exclusion of patients due to non-evaluable patients and the expectation of a 20% difference in outcomes (based on prior studies outside breast cancer) resulted in the study being underpowered to detect smaller differences in patient activation or SDM. Thus, this study is hypothesis generating only. Future studies in a larger sample are warranted to further elucidate the role of patient-reported data in decision-making.

## Conclusions

Oncologist engagement in the treatment planning process, with oncologist review of patient preferences and inclusion of patient-reported data in decision-making, is a promising approach to improve patient participation in treatment decisions. This approach may be particularly salient for certain subgroups of patients (e.g., Black women) who may face challenges to effectively communicating preferences during treatment discussion. Further trials are needed to assess the impact of this approach for broader and more diverse populations.

### Supplementary Information

Below is the link to the electronic supplementary material.

Supplementary file1 (DOCX 21 KB)

Supplementary file2 (DOCX 21 KB)

Supplementary file3 (DOCX 14 KB)

Supplementary file4 (DOCX 19 KB)

Supplementary file5 (DOCX 18 KB)

## Data Availability

De-identified data may be made available for interested investigators with IRB approval upon request.
