# Supplementary material for: A randomized controlled trial of shared decision-making treatment planning process to enhance shared decision-making in patients with MBC
Source: Breast Cancer Res Treat. 2024 Jun 10;206(3):483–93. doi: 10.1007/s10549-024-07304-y (PMC11208240; doi:10.1007/s10549-024-07304-y)
Supplement: Supplementary file 1 — Supplementary file1 (DOCX 21 KB) [file 10549_2024_7304_MOESM1_ESM.docx]

**Appendix A. Treatment Planning Survey - Questionnaire**

**Patient Information**

1. Who is completing the survey?
   - Patient (Myself)
   - Spouse or Partner
   - Adult child of the patient
   - Family member or relative (not the spouse or child)
   - Friend
   - Paid caretaker
   - Staff
   - Other
2. identify as? (select all that apply)

- American Indian/Alaskan Native
- Black/African American
- Native Hawaiian/Other Pacific Islander
- Asian
- White/Caucasian
- Hispanic/Latino
- Other (please specify)

**Health Literacy**

1. How often do you have someone help you read hospital materials? 
   Always

- Often
- Sometimes
- Occasionally
- Never

1. How often do you have problems learning about your medical condition because of difficulty understanding written information?

- Always
- Often
- Sometimes
- Occasionally
- Never

1. How often do you have a problem understanding what is told to you about your medical condition?

- Always
- Often
- Sometimes
- Occasionally
- Never

1. How confident are you filling out medical forms by yourself?

- Not at all
- A little bit
- Somewhat
- Quite a bit
- Extremely

1. Do you have an advance directive (a written document that describes the medical care you want in the future, or who you want to make decisions for you if you can’t make them for yourself)

- Yes
- No

1. Are you interested in learning about or participating in a clinical trial?

- Yes
- No

**Symptoms**

1. Over the past 2 weeks how often have you been bothered by the following problem?
2. Little interest or pleasure in doing things

- Not at all
- Several days
- More than half the days
- Nearly every day

1. Feeling down, depressed or helpless

- Not at all
- Several days
- More than half of the days
- Nearly every day

1. Trouble falling or staying asleep, or sleeping too much

- Not at all
- Several days
- More than half the days
- Nearly every day

1. Feeling tired or having little energy

- Not at all
- Several days
- More than half of the days
- Nearly every day

1. Poor appetite or overeating

- Not at all
- Several days
- More than half the days
- Nearly every day

1. Feeling bad about yourself, or that you are a failure or have let yourself or your family down

- Not at all
- Several days
- More than half the days
- Nearly every day

1. Trouble concentrating on things, such as reading the newspaper or watching television

- Not at all
- Several days
- More than half the days
- Nearly every day

1. Moving or speaking so slowly that other people could have noticed? Or the opposite- being so fidgety or restless that you have been moving around a lot more than usual

- Not at all
- Several days
- More than half the days
- Nearly every day

1. Thoughts that you would be better off dead or hurting yourself in some way

- Not at all
- Several days
- More than half the days
- Nearly every day

**Pain**

1. Please select the one number that best describes the PAIN you feel now (note: 0 = not experiencing the symptom to 10 = worst possible)
   1. **Pain Likert Scale (None 0 – 10 Worst Possible)**
2. Have you taken an opioid pain reliever (such as: oxycodone, morphine, hydrocodone, etc.) on a daily basis for the past week or more?

- Yes
- No

1. How do you describe your activity level within the past week?

0 - Normal with no limitations

1 - Not my normal self, but able to be up and about with fairly normal activities

2 - Not feeling up to most things, but in bed or chair less than half the day

3 - Able to do little activity & spend most of the day in bed or chair

4 - Pretty much bedridden, rarely out of bed

**NCCN Distress Thermometer**

Distress is an unpleasant emotional state that affects everyone with an illness at some time. It includes many feelings like sadness, worry, anger, helplessness and guilt. Distress can affect how you feel, think, and act.

1. How distressed are you feeling today? Select the number that describes your level of distress from 0 being the lowest and 10 being the highest distress you can imagine
   1. Overall Distress **(None 0 – 10 Worst Possible)**
2. Indicate if any of the following that has been a problem for you in the past week, including today
3. Practical Concerns: (select all that apply)

- Child care
- Housing
- Insurance or lack of insurance
- Financial or money
- Transportation
- Work/school
- None of these
- Other practical problems (please describe)__________________

1. Emotional Concerns: (select all that apply)

- Depression
- Fears
- Nervousness
- Sadness
- Worry
- Loss of interest in usual activities
- None of these
- Other emotional problems (please describe)_____________________

1. Family Concerns: (select all that apply)

- Dealing with children
- Resources for my caregiver
- Dealing with partner
- Family health issues
- None of these
- Other family problems (please describe)

1. Have spiritual or religious concerns been a problem for you in the past week, including today?

- I have spiritual or religious concerns
- I have no spiritual or religious concerns

1. Physical Symptoms Causing Concern: (select all that apply)

- Ability to have children
- Appearance
- Bathing/dressing
- Breathing
- Changes in urination
- Constipation
- Diarrhea
- Eating
- Fatigue
- Feeling swollen
- Fevers
- Getting around
- Indigestion
- Memory/concentration
- Mouth sores
- Nausea
- Nose dry/congested
- Pain
- Rash
- Sexual changes
- Skin changes
- Skin dry/itchy
- Sleep
- Substance abuse
- Tingling in hands/feet
- Taste Changes
- No Physical Problems
- Other physical problems (please describe) _______________________________Bottom of Form

**Referrals you may need**

1. Are you concerned about your ability to have children due to your cancer diagnosis?

- Yes
- No
- Does not apply to me

1. Have you ever had or are scheduled to have genetic testing done to see if you have inherited a gene that places you at a higher risk for developing cancer?

- Yes - within last 5 years
- Yes - longer than 5 years ago
- Yes - date of testing unknown
- No

1. How would you describe your use of tobacco (cigarettes)?

- Never smoked cigarettes
- Current cigarette smoker
- I am a former smoker for less than 1 year
- I have not smoked cigarettes for more than 1 year

1. (DEPENDENT QUESTION) Amount of tobacco used per day:
   - Infrequent or seldom
   - Less than 1 pack/day
   - More than 1 pack/day
