# Supplementary material for: A randomized controlled trial of shared decision-making treatment planning process to enhance shared decision-making in patients with MBC
Source: Breast Cancer Res Treat. 2024 Jun 10;206(3):483–93. doi: 10.1007/s10549-024-07304-y (PMC11208240; doi:10.1007/s10549-024-07304-y)
Supplement: Supplementary file 2 — Supplementary file2 (DOCX 21 KB) [file 10549_2024_7304_MOESM2_ESM.docx]

## Appendix B. Control Preferences Scale

**Physician**

Please tell me which statement best describes how your treatment decisions have been made:

1. The patient made the final decision about which treatment he/she would receive
2. The patient made the final decision about which treatment he/she would receive after considering my (the physician's) opinion
3. I (the physician) shared responsibility with the patient for making the final decision about treatment he/she would receive.
4. I (the physician) made the final decision about which treatment the patient would receive after seriously considering the patient's opinion.
5. I (the physician) made the final decision about which treatment the patient will receive.

**Patient**

Please tell me which statement best describes how your treatment decisions have been made:

1. I made the final selection about which treatment I would receive

2. I made the final selection of my treatment after seriously considering my doctor’s opinion.

3. My doctor and I shared responsibility for deciding which treatment was best for me.

4. My doctor made the final decision about which treatment will be used but seriously considered my opinion.

5. My doctor made all the decisions regarding my treatment.
