# Supplementary material for: A randomized controlled trial of shared decision-making treatment planning process to enhance shared decision-making in patients with MBC
Source: Breast Cancer Res Treat. 2024 Jun 10;206(3):483–93. doi: 10.1007/s10549-024-07304-y (PMC11208240; doi:10.1007/s10549-024-07304-y)
Supplement: Supplementary file 3 — Supplementary file3 (DOCX 14 KB) [file 10549_2024_7304_MOESM3_ESM.docx]

**Appendix C: Post-treatment decision physician questions.**

1. Please tell me which statement best describes how your treatment decisions have been made:
   1. The patient made the final decision about which treatment he/she would receive
   2. The patient made the final decision about which treatment he/she would receive after considering my (the physician's) opinion
   3. I (the physician) shared responsibility with the patient for making the final decision about treatment he/she would receive.
   4. I (the physician) made the final decision about which treatment the patient would receive after seriously considering the patient's opinion.
   5. I (the physician) made the final decision about which treatment the patient will receive.
2. Did you use the treatment plan in your conversation with the patient?
   1. Yes
   2. No
3. Did your management of the patient changed base on the patient-reported data?
   1. Yes
   2. No
4. The treatment plan helped me to engage in shared decision-making with my patient and the caregiver about treatment decisions.
   1. Strongly agree
   2. Agree
   3. Neutral
   4. Disagree
   5. Strongly Disagree
