# Supplementary material for: A randomized controlled trial of shared decision-making treatment planning process to enhance shared decision-making in patients with MBC
Source: Breast Cancer Res Treat. 2024 Jun 10;206(3):483–93. doi: 10.1007/s10549-024-07304-y (PMC11208240; doi:10.1007/s10549-024-07304-y)
Supplement: Supplementary file 4 — Supplementary file4 (DOCX 19 KB) [file 10549_2024_7304_MOESM4_ESM.docx]

**Supplemental Table 1.** Intention to Treat Analysis (N=141)

|  | | **Total (N=141)** | **Intervention (n=63)** | **Control (n=78)** | **Cramer's V** |
| --- | --- | --- | --- | --- | --- |
|  | | **n (%)** | | |  |
| **Age at consent Median (IQR)** | | 58.2 (12.4) | 56.6 (13.2) | 59.5 (11.6) | Cohen's d = -0.234 |
| **Race/Ethnicity** | |  |  |  | 0.104 |
|  | Asian | 2 (1.4) | 1 (1.6) | 1 (1.3) |  |
|  | Black | 39 (27.7) | 16 (25.4) | 23 (29.5) |  |
|  | Hispanic/Latino | 1 (0.7) | 1 (1.6) | 0 |  |
|  | White | 95 (67.4) | 43 (68.3) | 52 (66.7) |  |
|  | Declined | 4 (2.8) | 2 (3.2) | 2 (2.6) |  |
| **Area Deprivation Index (ADI)** | |  |  |  | 0.116 |
|  | Least Distance | 75 (53.2) | 35 (55.6) | 40 (51.3) |  |
|  | Most Distance | 16 (11.4) | 9 (14.3) | 7 (9.0) |  |
|  | Unknown | 50 (35.5) | 19 (30.2) | 31 (39.7) |  |
| **Rural-Urban Commuting Area (RUCA)** | | |  |  | 0.111 |
|  | Rural | 32 (22.7) | 17 (27.0) | 15 (19.2) |  |
|  | Urban | 94 (66.7) | 41 (65.1) | 53 (68.0) |  |
|  | Unknown | 15 (10.6) | 5 (7.9) | 10 (12.8) |  |
| **Median time traveled (IQR)** | | 59.0 (27.5-99.0) | 56.5 (28.0-99.0) | 61.0 (27.0-99.0) | Kendall τb=0.933 |
| **Median distance traveled (in miles)** | | 57.6 (20.5-99.8) | 56.7 (21.4-95.6) | 57.6 (19.0-101.0) | Kendall τb=0.926 |
| **Insurance status** | |  |  |  | 0.037 |
|  | Private | 46 (32.6) | 20 (31.8) | 26 (33.3) |  |
|  | Medicaid | 14 (9.9) | 7 (11.1) | 7 (9.0) |  |
|  | Medicare | 81 (57.5) | 36 (31.8) | 45 (57.7) |  |
| **Cancer Subtype** | |  |  |  | 0.186 |
|  | HR+HER2+ | 24 (17.7) | 8 (12.7) | 17 (21.8) |  |
|  | HR+HER2- | 97 (68.8) | 46 (73.0) | 51 (65.4) |  |
|  | HR-HER2+ | 5 (3.6) | 4 (6.4) | 1 (1.3) |  |
|  | TNBC | 14 (9.9) | 5 (7.9) | 9 (11.5) |  |
| **Type of MBC** | |  |  |  | 0.108 |
|  | De novo | 14 (9.9) | 4 (6.4) | 10 (12.8) |  |
|  | Recurrent | 127 (90.1) | 59 (93.7) | 68 (87.2) |  |
| **Patients desire for clinical trials** | | |  |  | 0.081 |
|  | Yes | 116 (82.3) | 54 (85.7) | 62 (79.5) |  |
|  | No | 25 (17.7) | 9 (14.3) | 16 (20.5) |  |
| **Baseline Control Preference Scale (Carevive)** | | |  |  | 0.126 |
|  | Oncologist-centric | 10 (7.1) | 5 (6.4) | 5 (7.9) |  |
|  | Shared | 81 (57.5) | 48 (61.5) | 33 (52.4) |  |
|  | Patient-centric | 49 (34.8) | 25 (32.1) | 24 (38.1) |  |
|  | N/A | 1 (0.7) | 0 | 1 (1.6) |  |
| **Patient Control Preference Scale (post-intervention)** | | | |  | 0.098 |
|  | Oncologist-centric | 33 (23.4) | 14 (22.2) | 19 (24.4) |  |
|  | Shared | 78 (55.3) | 38 (60.3) | 40 (51.3) |  |
|  | Patient-centric | 30 (21.3) | 11 (17.5) | 19 (24.4) |  |
| **Oncologist Control Preference Scale** | | |  |  | 0.176 |
|  | Oncologist-centric | 46 (32.6) | 19 (30.2) | 27 (34.6) |  |
|  | Shared | 97 (61.7) | 43 (68.3) | 7 (9.0) |  |
|  | Patient-centric | 8 (5.7) | 1 (1.6) | 44 (56.4) |  |
| **Patient Activation Measure** | |  |  |  | 0.215 |
|  | Level 1 | 9 (6.4) | 1 (1.6) | 8 (10.3) |  |
|  | Level 2 | 20 (14.2) | 8 (12.7) | 12 (15.4) |  |
|  | Level 3 | 61 (43.3) | 26 (41.3) | 35 (44.9) |  |
|  | Level 4 | 51 (36.2) | 28 (44.4) | 23 (29.5) |  |
| **Treatment Satisfaction Questionnaire** | | |  |  |  |
|  | Effectiveness scale | 47.2 (12.6) | 48.2 (13.9) | 46.4 (11.4) | Cohen's d = 0.14 |
|  | Side effects scale | 38.9 (18.5) | 40.0 (17.6) | 37.8 (19.5) | Cohen's d = 0.12 |
|  | Convenience scale | 54.8 (14.8) | 57.3 (15.8) | 52.7 (13.7) | Cohen's d = 0.31 |
|  | Global Satisfaction scale | 44.8 (16.1) | 44.6 (17.6) | 44.9 (14.9) | Cohen's d = -0.02 |
| HR: Hormone Receptor; TNBC: triple-negative breast cancer; IQR: interquartile range; MBC: MBC; N/A: not applicable; HER2+: human epidermal growth factor receptor 2. | | | | | |
