# Supplementary material for: A randomized controlled trial of shared decision-making treatment planning process to enhance shared decision-making in patients with MBC
Source: Breast Cancer Res Treat. 2024 Jun 10;206(3):483–93. doi: 10.1007/s10549-024-07304-y (PMC11208240; doi:10.1007/s10549-024-07304-y)
Supplement: Supplementary file 5 — Supplementary file5 (DOCX 18 KB) [file 10549_2024_7304_MOESM5_ESM.docx]

**Supplemental Table 2.** Demographic and clinical characteristics of patients excluded from study (n=32)

|  | **Total**  **(N=32)** | **Intervention (n=11)** | **Control (n=21)** | **Cramer's V** |
| --- | --- | --- | --- | --- |
|  | n (%) | | |  |
| **Age at consent Median (IQR)** | 65.5 (56.0-71.5) | 68.0 (58.0-72.0) | 63.0 (40.0-68.0) | Glass's delta = 0.351 |
| **Race and Ethnicity** |  |  |  | 0.085 |
| Asian | 0 | 0 | 0 |  |
| Black | 9 (28.1) | 3 (27.3) | 6 (28.6) |  |
| Hispanic/Latino | 0 | 0 | 0 |  |
| White | 21 (65.6) | 7 (63.6) | 14 (66.7) |  |
| Declined | 2 (6.3) | 1 (9.1) | 1 (4.8) |  |
| **Area Deprivation Index (ADI)** |  |  |  | 0.256 |
| Least Distance | 18 (56.3) | 8 (72.7) | 10 (47.6) |  |
| Most Distance | 3 (9.4) | 1 (9.1) | 2 (9.5) |  |
| Unknown | 11 (34.4) | 2 (18.2) | 9 (42.9) |  |
| **Rural-Urban Commuting Area (RUCA)** |  |  |  | 0.333 |
| Rural | 6 (18.8) | 4 (36.4) | 2 (9.5) |  |
| Urban | 21 (65.6) | 6 (54.6) | 15 (71.4) |  |
| Unknown | 5 (15.6) | 1 (9.1) | 4 (19.1) |  |
| **Median time traveled (in minutes) (IQR)** | 51.0 (22.0-99.0) | 80.5 (48.0-135.0) | 32.0 (22.0-88.0) | Kendall τb = -0.313 |
| **Median distance traveled (in miles) Median (IQR)** | 43.8 (13.2-103.0) | 71.5 (43.6-140.0) | 24.1 (13.2-80.1) | Kendall τb = -0.331 |
| **Insurance status** |  |  |  | 0.323 |
| Private | 7 (21.9) | 2 (18.2) | 5 (23.8) |  |
| Medicaid | 4 (12.5) | 3 (27.3) | 1 (4.8) |  |
| Medicare | 21 (65.6) | 6 (54.6) | 15 (71.4) |  |
| **Cancer subtype** |  |  |  | 0.287 |
| HR+HER2+ | 3 (9.4) | 1 (9.1) | 2 (9.5) |  |
| HR+HER2- | 24 (75.0) | 7 (63.6) | 17 (81.0) |  |
| HR-HER2+ | 1 (3.1) | 1 (9.1) | 0 |  |
| TNBC | 4 (12.5) | 2 (18.2) | 2 (9.5) |  |
| **Type of Metastatic Breast Cancer (MBC)** |  |  |  |  |
| De novo | 4 (19.1) | 2 (18.2) | 4 (19.1) | 0.011 |
| Recurrent | 17 (81.0) | 9 (81.8) | 17 (81.0) |  |
| **Median time from diagnosis of MBC to Treatment Decision (in months) (IQR)** | 4.5 (0-20.0) | 11.0 (1.0-24.0) | 2.0 (0-13.0) | Kendall τb = -0.197 |
| **Patients desire for clinical trials** |  |  |  | 0.209 |
| Yes | 24 (75.0) | 10 (90.9) | 14 (66.7) |  |
| No | 8 (25.0) | 1 (9.1) | 7 (33.3) |  |
| **Baseline Control Preference Scale  (Carevive)** |  |  |  | 0.249 |
| Oncologist-centric | 1 (3.1) | 1 (9.1) | 0 |  |
| Shared | 19 (59.4) | 6 (54.6) | 13 (61.9) |  |
| Patient-centric | 12 (37.5) | 4 (36.4) | 8 (38.1) |  |
| HR: Hormone Receptor; TNBC: triple-negative breast cancer; IQR: interquartile range; MBC: metastatic breast cancer; N/A: not applicable; HER2+: human epidermal growth factor receptor 2. | | | | |
